# Allocative inefficiency in physician workforce distribution: specialty-level NHI-covered reimbursement analysis in South Korea’s single-payer system

**DOI:** 10.3389/fpubh.2026.1878385

**Published:** 2026-08-12

**Authors:** Sun-Cheol Kim, Je-Il Ryu

**Affiliations:** 1 Graduate School of Business, Hanyang University, Seoul, Republic of Korea; 2 Department of Neurosurgery, Hanyang University Guri Hospital, Hanyang University College of Medicine, Guri, Republic of Korea

**Keywords:** allocative inefficiency, health policy, health workforce, national health insurance, physician incentive plans, Republic of Korea, residency training, single-payer system

## Abstract

**Background:**

About 52 million residents are insured under the single-payer National Health Insurance (NHI) of South Korea. Twenty-six board-certified clinical specialties practice under this system, yet specialty-level matching between physician manpower and NHI-financed workload has not been reported.

**Methods:**

This study is an ecological cross-sectional analysis using specialty (*n* = 26) as the unit of observation. For 2023 and 2024, annual NHI reimbursement per specialty was divided by the mean specialist headcount to yield per-specialist reimbursement. This indicator captures facility-level resource use rather than physician effort in isolation. Specialties were placed on a reimbursement × application-rate plane and sorted into four quadrants. Inter-specialty reallocation was simulated at 100, 70, and 50% efficiency.

**Results:**

Per-specialist reimbursement was 52.5 million KRW (approximately USD 40,000) in radiology (*n* = 4,247) and 2,668.6 million KRW (approximately USD 2.04 million) in radiation oncology (*n* = 330). The resulting ratio was 50.8-fold. Coefficient of variation exceeded 90% in both years. Five specialties combined above-median reimbursement with sub-100% application rates; seven showed the reverse pattern. Utilization-volume triangulation confirmed that per-specialist reimbursement was strongly correlated with claims per specialist (Spearman *ρ* = 0.74) and patients per specialist (*ρ* = 0.66), supporting its interpretation as a workload-related indicator. The rank-order association between reimbursement and application rate was not statistically detectable (*ρ* = 0.180; 95% confidence interval, −0.24 to 0.54; *p* = 0.400), and the study was underpowered for this secondary test. A counterfactual calculation, presented as an upper-bound illustration rather than a policy projection, indicated that relocating 100 specialists from lower-reimbursement to higher-reimbursement quadrants at 70% efficiency corresponded to 68.8 billion KRW in annual NHI reimbursement.

**Conclusion:**

Across 2 study years, the 50.8-fold gap between these two specialties did not narrow. This dispersion, which reflects NHI-financed resource consumption rather than physician effort alone, is the principal finding. A statistically detectable link between trainee competition and NHI reimbursement was not observed; the secondary correlation test was underpowered, and its null result is not evidence of no association. These findings provide a specialty-level reimbursement baseline for South Korea’s workforce allocation debate.

## 1 Introduction

South Korea runs a single-payer National Health Insurance (NHI) system. About 52 million beneficiaries are enrolled, and providers receive payment through a uniform fee-for-service schedule (1). Whether the 26 board-certified clinical specialties recognized under this system receive physician labor in proportion to their NHI-financed patient volumes remains contested (2, 3). In this study, allocative efficiency denotes the degree to which the physician workforce of each specialty is aligned with its NHI-financed service volume; allocative inefficiency denotes misalignment between the two. In February 2024, an estimated 12,000 trainee physicians, roughly 93% of the national resident workforce, resigned from their hospital posts after the government disclosed plans to add 2,000 seats to medical school admissions beginning in 2025 (4, 5). The event exposed a long-standing tension between two contrasting positions: projections of aggregate physician shortage by 2035, and evidence that graduating physicians cluster in a narrow band of specialties, leaving surgical and emergency disciplines chronically understaffed (6–8).

Three methodological traditions have addressed this question, none with claims-level NHI data disaggregated to the specialty level. System dynamics models, most recently a regional compartment framework, have projected total physician headcounts without distinguishing among specialties (6). Cross-sectional surveys of medical students (sample sizes typically 200–500) have catalogued stated preferences for lifestyle-friendly disciplines (9, 10). Policy analyses grounded in Kingdon’s multiple-streams model have described, qualitatively, the avoidance of essential specialties (11). A recent analysis published in J Korean Med Sci computed total medical expenditures per active physician at the national level; that ratio, pooling all 26 specialties into a single denominator, could not reveal inter-specialty variation (12). To date, based on a PubMed and KoreaMed search conducted in January 2026, published analyses have not yet decomposed NHI-covered reimbursement on a per-specialist basis across all 26 recognized clinical specialties or linked such figures to residency recruitment patterns in a structured two-axis framework.

This study pursued three objectives. First, to calculate NHI-covered reimbursement per specialist across all 26 clinical specialties, with separate estimates for fiscal year 2023 (pre-crisis baseline) and fiscal year 2024 (crisis year); this metric captures NHI-financed resource consumption per specialist rather than physician labor alone. Second, to position each specialty on a two-axis scatter plot (reimbursement versus residency competition) and classify it into one of four quadrants. Third, to model the NHI output change from a hypothetical inter-specialty reallocation of 50 to 500 specialists, applying efficiency assumptions of 100, 70, and 50% to approximate retraining losses.

## 2 Materials and methods

### 2.1 Study design and data sources

In the South Korean training system, medical graduates enter a specialty through an annual first-round application to hospital residency posts, and the ratio of applicants to posted positions defines the application rate used in this study. Residency lasts three to 4 years depending on the specialty and leads to board certification by the relevant specialty society. Certified specialists then practice across five facility tiers, from tertiary hospitals to clinics, and their services are reimbursed under a uniform NHI fee schedule. This ecological cross-sectional study used the medical specialty (*n* = 26) as its unit of analysis. Both study years (2023 and 2024) fell within South Korea’s NHI claims reporting cycle. All variables were derived from publicly released aggregate tabulations; no patient- or physician-level identifiers were accessed. NHI reimbursement data came from the Health Insurance Review and Assessment Service (HIRA) open-data portal hosted at data.go.kr (13). Each annual file contained, for every combination of facility tier (5 tiers: tertiary hospital, general hospital, hospital, long-term care hospital, clinic) and clinical specialty, the summed NHI-covered reimbursement in Korean won (KRW), the number of unique patients, and the number of insurance claims. Specialist headcounts, broken down by specialty and facility tier, were obtained from the HIRA Medical Resources Statistics portal at opendata.hira.or.kr (14). HIRA publishes these counts quarterly (March, June, September, December); the four values within each calendar year were averaged to yield a single annual figure per specialty. Residency application data (first-round applicant counts and posted positions, stratified by specialty) were obtained from Ministry of Health and Welfare press releases (early 2023, early 2024) (15). Population figures by province (5-year age bands) and regional per-capita gross domestic product data were retrieved from the Korean Statistical Information Service (16, 17). Given only 26 specialty-level observations, entering them into a multivariable regression would risk overfitting; they were therefore excluded from the analytic model.

### 2.2 Variable construction

For each of the 26 specialties, a single metric, designated “NHI-covered reimbursement per specialist,” served as the primary outcome, computed as the total NHI reimbursement across 5 facility tiers for each specialty divided by the mean annual specialist count for that specialty. Revenue outside the NHI fee schedule was not included in the numerator. Cosmetic dermatological procedures, aesthetic plastic operations, and elective refractive eye surgery fall into this excluded category. This indicator should be read as NHI-financed resource consumption per specialist; it does not isolate physician labor from capital and facility-tier inputs. HIRA assigns reimbursement at the departmental level rather than the individual-physician level, so claims from support services such as anesthesiology, pathology, and radiology are attributed to the procedural department; this attribution inflates the reimbursement of procedural specialties and understates that of support specialties. Separately, the residency application rate (first-round applicants divided by posted positions, by specialty) quantified how many candidates competed for each training slot (15). Two additional per-specialist volume indicators were derived from the same annual files: the number of unique patients per specialist and the number of insurance claims per specialist. A per-claim indicator (reimbursement divided by claims) was also computed. These volume measures were used to triangulate whether per-specialist reimbursement reflects clinical workload rather than fee weighting alone.

### 2.3 Analytical framework

**Figure 1:**
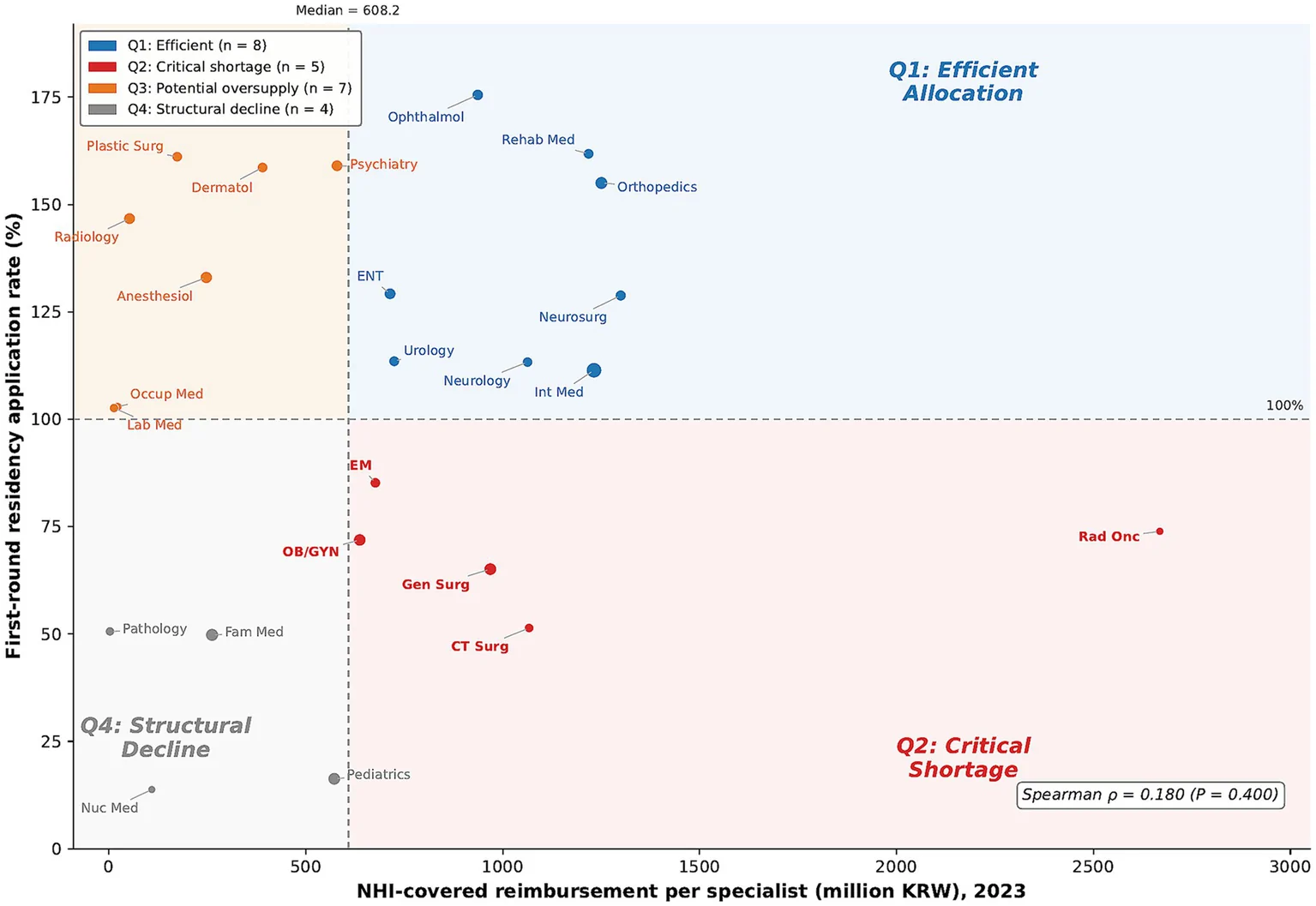
Scatter plot of NHI-covered reimbursement per specialist (*x*-axis, million KRW) versus residency application rate (*y*-axis, %) for 24 clinical specialties in 2023. The vertical dashed line indicates the median NHI-covered reimbursement across 26 specialties. The horizontal dashed line marks the 100% application rate threshold. Four quadrants are defined: Q1 (above-median reimbursement, application rate ≥100%), Q2 (above-median reimbursement, application rate <100%), Q3 (below-median reimbursement, application rate ≥100%), and Q4 (below-median reimbursement, application rate <100%). Two specialties without residency programs (tuberculosis, preventive medicine) were excluded from the plot. NHI, National Health Insurance; KRW, Korean won.

Analysis had three phases. Phase 1 tabulated per-specialist NHI-covered reimbursement for all 26 specialties in 2023 and 2024, ranked them, computed year-on-year percentage changes, and summarized inter-specialty dispersion via the coefficient of variation (CV). Phase 2 applied a two-by-two classification analogous to the growth-share matrix used in strategic management (18), in which two continuous axes are dichotomized to produce four interpretive cells; to the authors’ knowledge this is the first application of such a matrix to specialty-level workforce allocation. Allocative efficiency was operationalized as the alignment between a specialty’s NHI-financed workload and its trainee recruitment: alignment (high reimbursement with high fill, or low reimbursement with low fill) versus misalignment (the two off-diagonal cells). This operational definition is narrower than the welfare-economic concept of allocative efficiency, which requires resources to be allocated according to marginal benefit. Per-specialist reimbursement was placed on the *x*-axis and residency application rate on the *y*-axis, and the plane was divided into quadrants by two cut-points. The cut-point on the reimbursement axis was set at the median of the 26 specialties, drawn as the vertical dashed line in Figure 1; the cut-point on the application-rate axis was fixed at 100%, drawn as the horizontal dashed line, representing exact filling of the posted positions. Four cells resulted. Q1 grouped specialties with above-median reimbursement and an application rate of 100% or higher (efficient allocation). Q2 referred to above-median reimbursement combined with an application rate below 100% (critical shortage), whereas Q3 included below-median reimbursement with an application rate of 100% or higher (potential oversupply). Q4 was defined as below-median reimbursement together with a sub-100% application rate (structural decline). The rank-order association between the two axes was tested by Spearman’s *ρ*. Between Phases 2 and 3, the rank-order correlation between per-specialist reimbursement and each volume indicator (patients per specialist, claims per specialist) was computed by Spearman’s *ρ* to test the workload interpretation of the reimbursement metric. Phase 3 ran a counterfactual exercise: 50, 100, 200, or 500 specialists were hypothetically moved from the average reimbursement of Q3/Q4 specialties to that of Q2 specialties. Three efficiency discounts (100, 70, 50%) were applied to reflect retraining costs and learning-curve attrition.

### 2.4 Statistical analysis

Descriptive results are reported as means, medians, and CVs. Spearman’s *ρ* was chosen over Pearson’s r because the 26-specialty sample could not be assumed to follow a bivariate normal distribution. Excluding three high non-NHI-revenue specialties (dermatology, plastic surgery, ophthalmology) tested whether those outliers drove the overall estimate. Because each candidate may list only one specialty in the first round, application rates above 100% indicate that first-round applicants exceeded posted positions for that specialty, and rates below 100% indicate unfilled posts after the first round; later recruitment rounds were not included. Python 3.11 with SciPy 1.11 performed all computations. Two-tailed *p* < 0.05 defined significance. United States dollar equivalents were calculated at the 2023 average exchange rate of 1,305 KRW per USD.

### 2.5 Ethics statement

Only de-identified, aggregate-level public statistics were analyzed. No institutional review board review was sought; informed consent did not apply.

## 3 Results

### 3.1 Specialty-level NHI-covered reimbursement

Across the 26 specialties, 95,450 specialists were registered in 2023 and 97,075 in 2024, a net gain of 1,625 (1.7%). Total NHI reimbursement rose from 74.0 trillion KRW in 2023 to 77.2 trillion KRW in 2024 (Table 1). Per-specialist reimbursement spanned 2.8 million KRW in pathology (*n* = 963) to 2,668.6 million KRW in radiation oncology (*n* = 330). The four lowest values, in pathology, laboratory medicine, occupational and environmental medicine, and preventive medicine, chiefly reflect the departmental attribution of support-service claims described in Section 2.2; their annual claim volumes per specialist were below 120, against a 26-specialty median of approximately 3,600 (Table 2). Radiology (52.5 million KRW; *n* = 4,247), the lowest value outside this group and itself reduced by the same attribution rule, stood 50.8-fold below radiation oncology. Five specialties crossed 1,200 million KRW: radiation oncology (2,669; *n* = 330), neurosurgery (1,300; *n* = 3,071), orthopedic surgery (1,251; *n* = 7,063), internal medicine (1,232; *n* = 18,366), and rehabilitation medicine (1,218; *n* = 2,462). Plastic surgery (173.6; *n* = 2,347) and anesthesiology (247.5; *n* = 5,295) remained near the bottom. CV: 92.8% (2023), 94.1% (2024). Inter-specialty dispersion did not narrow.

**Table 1:** NHI-covered reimbursement per specialist by specialty, 2023 and 2024.

Rank	Specialty	n 2023	NHI 2023	n 2024	NHI 2024	Δ NHI	Δ %
1	Radiation oncology	330	2,668.6	339	2,782.7	114.1	4.3
2	Neurosurgery	3,071	1,299.7	3,139	1,325.1	25.4	2.0
3	Orthopedic surgery	7,063	1,250.6	7,217	1,307.7	57.1	4.6
4	Internal medicine	18,366	1,231.9	18,659	1,230.0	−1.9	−0.2
5	Rehabilitation medicine	2,462	1,218.2	2,552	1,293.6	75.4	6.2
6	Cardiothoracic surgery	1,169	1,067.0	1,182	1,157.0	90.0	8.4
7	Neurology	2,141	1,063.2	2,200	1,047.2	−16.0	−1.5
8	General surgery	6,651	968.6	6,691	1,025.7	57.1	5.9
9	Ophthalmology	3,733	936.7	3,800	971.8	35.1	3.7
10	Urology	2,644	724.6	2,648	763.1	38.5	5.3
11	Otorhinolaryngology	4,202	714.1	4,264	683.4	−30.7	−4.3
12	Emergency medicine	2,301	676.7	2,424	581.3	−95.4	−14.1
13	OB/GYN	6,035	637.1	6,049	708.7	71.6	11.2
14	Psychiatry	4,102	579.4	4,221	599.9	20.5	3.5
15	Pediatrics	6,355	572.4	6,466	617.5	45.1	7.9
16	Dermatology	2,442	390.3	2,483	404.3	14.0	3.6
17	Family medicine	7,390	262.1	7,477	265.2	3.1	1.2
18	Anesthesiology	5,295	247.5	5,401	260.5	13.0	5.3
19	Plastic surgery	2,347	173.6	2,386	158.7	−14.9	−8.6
20	Nuclear medicine	260	109.1	263	116.7	7.6	7.0
21	Tuberculosis	54	99.8	57	86.3	−13.5	−13.5
22	Radiology	4,247	52.5	4,313	53.5	1.0	1.9
23	Preventive medicine	192	38.5	196	34.7	−3.8	−9.9
24	Occupational & environmental medicine	648	22.2	665	16.7	−5.5	−24.8
25	Laboratory medicine	987	13.3	1,011	6.5	−6.8	−51.1
26	Pathology	963	2.8	972	3.4	0.6	21.4

NHI, National Health Insurance; n, number of specialists; M KRW, million Korean won; OB/GYN, obstetrics and gynecology. Reimbursement values are expressed as M KRW and computed as total NHI reimbursement across 5 facility tiers ÷ mean annual specialist count. Ranked by 2023 reimbursement (descending). Two specialties without residency programs (tuberculosis, preventive medicine) are included here but were excluded from quadrant analysis.

**Table 2:** Utilization-volume triangulation of per-specialist reimbursement, patients, claims, and reimbursement per claim, 2023 and 2024.

Specialty	Reimb./spec. 2023	Patients/spec. 2023	Claims/spec. 2023	Reimb./claim 2023	Reimb./spec. 2024	Patients/spec. 2024	Claims/spec. 2024	Reimb./claim 2024
Radiation oncology	2,668.6	484	5,500	485.2	2,782.7	489	5,392	516.1
Neurosurgery	1,299.7	1,763	6,557	198.2	1,325.1	1,718	6,475	204.7
Orthopedic surgery	1,250.6	2,841	12,525	99.8	1,307.7	2,783	12,480	104.8
Internal medicine	1,231.9	1,984	9,531	129.3	1,230.0	1,909	9,323	131.9
Rehabilitation medicine	1,218.2	907	5,504	221.3	1,293.6	896	5,534	233.8
Cardiothoracic surgery	1,067.0	490	1,464	728.7	1,157.0	467	1,434	806.9
Neurology	1,063.2	1,699	5,748	185.0	1,047.2	1,673	5,666	184.8
General surgery	968.6	994	3,355	288.7	1,025.7	979	3,307	310.1
Ophthalmology	936.7	4,303	10,878	86.1	971.8	4,146	10,544	92.2
Urology	724.6	2,026	6,682	108.4	763.1	2,031	6,715	113.6
Otorhinolaryngology	714.1	5,278	19,502	36.6	683.4	5,010	18,348	37.2
Emergency medicine	676.7	2,152	2,864	236.3	581.3	1,602	2,094	277.6
OB/GYN	637.1	1,168	3,932	162.0	708.7	1,144	3,924	180.6
Psychiatry	579.4	737	5,435	106.6	599.9	746	5,579	107.5
Pediatrics	572.4	1,709	12,035	47.6	617.5	1,537	11,094	55.7
Dermatology	390.3	3,376	9,510	41.0	404.3	3,274	9,320	43.4
Family medicine	262.1	659	2,619	100.1	265.2	612	2,503	106.0
Anesthesiology	247.5	693	3,181	77.8	260.5	689	3,234	80.6
Plastic surgery	173.6	252	819	211.9	158.7	238	790	200.8
Nuclear medicine	109.1	166	496	219.9	116.7	162	486	240.2
Tuberculosis	99.8	78	340	293.6	86.3	61	336	256.7
Radiology	52.5	210	305	172.3	53.5	206	301	177.5
Preventive medicine	38.5	30	85	454.5	34.7	24	79	441.8
Occupational & environmental medicine	22.2	48	114	194.4	16.7	40	97	171.7
Laboratory medicine	13.3	57	96	139.2	6.5	9	30	213.4
Pathology	2.8	4	6	454.5	3.4	1	3	1,136.8

Reimb./spec., NHI reimbursement per specialist (million KRW); Patients/spec., unique patients per specialist; Claims/spec., insurance claims per specialist; Reimb./claim, reimbursement per claim (thousand KRW). Reimbursement per specialist values are identical to those in Table 1; patient and claim counts were tabulated from the same HIRA annual files, and reimbursement per claim was derived from these two columns. Ranked by 2023 reimbursement per specialist (descending). Spearman ρ between reimbursement per specialist and claims per specialist was 0.74 (2023) and 0.73 (2024); with patients per specialist, 0.66 (2023) and 0.67 (2024); all *p* < 0.001.

### 3.2 Utilization-volume triangulation

Two volume indicators were examined alongside per-specialist reimbursement (Table 2). Per-specialist reimbursement was strongly correlated with claims per specialist (Spearman *ρ* = 0.74 in 2023 and 0.73 in 2024; both *p* < 0.001) and with patients per specialist (*ρ* = 0.66 in 2023 and 0.67 in 2024; both *p* < 0.001), indicating that the reimbursement metric tracks clinical volume across specialties rather than fee weighting alone. The dispersion seen in reimbursement was also present in the volume measures: the coefficient of variation was 99.2% for claims per specialist and 105.6% for patients per specialist in 2023, comparable to the 92.8% for reimbursement per specialist. Two capital-intensive specialties departed from the general pattern. Radiation oncology ranked first in reimbursement per specialist but eleventh in claims per specialist, with the second-highest reimbursement per claim (485,200 KRW), and cardiothoracic surgery ranked sixth in reimbursement per specialist but eighteenth in claims per specialist, with the highest reimbursement per claim in 2023 (728,700 KRW); in both, the high per-specialist reimbursement reflected the value of each service rather than a high service volume. Radiology and pathology ranked in the lowest quartile on every volume measure, consistent with the departmental attribution of their claims to procedural specialties described in the Methods.

### 3.3 Year-on-year changes: 2023 versus 2024

Most specialties held their 2023 ranks in 2024 (Table 3). The top five did not change. Per-specialist reimbursement of obstetrics and gynecology (OB/GYN) rose 11.2% (637.1 to 708.7 million KRW) with almost no headcount change (6,035 to 6,049 specialists). Cardiothoracic surgery and pediatrics also grew, by 8.4 and 7.9%. Emergency medicine showed an opposite trend. Even with 123 new specialists (2,301 to 2,424), its per-specialist figure fell 14.1%, from 676.7 to 581.3 million KRW. This simultaneous drop in reimbursement and rise in headcount fits the reduction of emergency department volume reported for February through December 2024, the trainee walkout period (5).

**Table 3:** Year-on-year changes in NHI-covered reimbursement per specialist, 2023 to 2024.

Rank 2023	Specialty	Rank 2024	NHI 2023	NHI 2024	Δ n	Δ NHI	Δ %
1	Radiation oncology	1	2,668.6	2,782.7	9	114.1	4.3
2	Neurosurgery	2	1,299.7	1,325.1	68	25.4	2.0
3	Orthopedic surgery	3	1,250.6	1,307.7	154	57.1	4.6
4	Internal medicine	5	1,231.9	1,230.0	293	−1.9	−0.2
5	Rehabilitation medicine	4	1,218.2	1,293.6	90	75.4	6.2
6	Cardiothoracic surgery	6	1,067.0	1,157.0	13	90.0	8.4
7	Neurology	7	1,063.2	1,047.2	59	−16.0	−1.5
8	General surgery	8	968.6	1,025.7	40	57.1	5.9
9	Ophthalmology	9	936.7	971.8	67	35.1	3.7
10	Urology	10	724.6	763.1	4	38.5	5.3
11	Otorhinolaryngology	12	714.1	683.4	62	−30.7	−4.3
12	Emergency medicine	15	676.7	581.3	123	−95.4	−14.1
13	OB/GYN	11	637.1	708.7	14	71.6	11.2
14	Psychiatry	14	579.4	599.9	119	20.5	3.5
15	Pediatrics	13	572.4	617.5	111	45.1	7.9
16	Dermatology	16	390.3	404.3	41	14.0	3.6
17	Family medicine	17	262.1	265.2	87	3.1	1.2
18	Anesthesiology	18	247.5	260.5	106	13.0	5.3
19	Plastic surgery	19	173.6	158.7	39	−14.9	−8.6
20	Nuclear medicine	20	109.1	116.7	3	7.6	7.0
21	Tuberculosis	21	99.8	86.3	3	−13.5	−13.5
22	Radiology	22	52.5	53.5	66	1.0	1.9
23	Preventive medicine	23	38.5	34.7	4	−3.8	−9.9
24	Occupational & environmental medicine	24	22.2	16.7	17	−5.5	−24.8
25	Laboratory medicine	25	13.3	6.5	24	−6.8	−51.1
26	Pathology	26	2.8	3.4	9	0.6	21.4

Reimbursement values in million Korean won (M KRW); OB/GYN, obstetrics and gynecology. Sorted by 2023 rank. Δ n = net change in specialist headcount. Δ % = percentage change in per-specialist reimbursement.

### 3.4 Reimbursement–competition mismatch quadrant

Of the 24 specialties with residency programs, 8 fell in Q1, 5 in Q2, 7 in Q3, and 4 in Q4 in 2023 (Table 4; Figure 1). The 2024 distribution was identical at the aggregate level, though two individual specialties crossed quadrant boundaries. Q2 (high reimbursement, low competition) in 2023: radiation oncology (2,669 million KRW; 73.9% application rate), cardiothoracic surgery (1,067; 51.4%), general surgery (968.6; 65.1%), emergency medicine (676.7; 85.2%), OB/GYN (637.1; 71.9%). Q3 (low reimbursement, high competition): psychiatry (579.4; 159.0%), dermatology (390.3; 158.6%), anesthesiology (247.5; 133.0%), plastic surgery (173.6; 161.1%), radiology (52.5; 146.7%), among others. Between the two years, pediatrics moved from Q4 to Q2: reimbursement climbed from 572.4 to 617.5 million KRW (above the median), but its first-round application rate was only 25.9%. Emergency medicine moved from Q2 to Q4 after its reimbursement dropped below the 2024 median. Spearman’s *ρ* between NHI-covered reimbursement and application rate: 0.180 [95% confidence interval (CI), −0.24 to 0.54; *p* = 0.400] in 2023; 0.101 (95% CI, −0.32 to 0.48; *p* = 0.639) in 2024 (Table 5). Neither value reached the 0.05 threshold. Excluding dermatology, plastic surgery, and ophthalmology raised ρ to 0.281 (*p* = 0.218), still non-significant.

**Table 4:** Reimbursement–competition quadrant classification of 24 clinical specialties, 2023.

Quadrant	Specialty	Specialists	Reimbursement (M KRW)	Application rate (%)
Q1 (High reimb., ≥100% fill): Efficient allocation	Neurosurgery	3,071	1,299.7	128.8
	Orthopedic surgery	7,063	1,250.6	155.0
	Internal medicine	18,366	1,231.9	111.4
	Rehabilitation medicine	2,462	1,218.2	161.8
	Neurology	2,141	1,063.2	113.3
	Ophthalmology	3,733	936.7	175.5
	Urology	2,644	724.6	113.5
	Otorhinolaryngology	4,202	714.1	129.2
Q2 (High reimb., <100% fill): Critical shortage	Radiation oncology	330	2,668.6	73.9
	Cardiothoracic surgery	1,169	1,067.0	51.4
	General surgery	6,651	968.6	65.1
	Emergency medicine	2,301	676.7	85.2
	OB/GYN	6,035	637.1	71.9
Q3 (Low reimb., ≥100% fill): Potential oversupply	Psychiatry	4,102	579.4	159.0
	Dermatology	2,442	390.3	158.6
	Anesthesiology	5,295	247.5	133.0
	Plastic surgery	2,347	173.6	161.1
	Radiology	4,247	52.5	146.7
	Occupational & environmental medicine	648	22.2	102.9
	Laboratory medicine	987	13.3	102.6
Q4 (Low reimb., <100% fill): Structural decline	Pediatrics	6,355	572.4	16.3
	Family medicine	7,390	262.1	49.8
	Nuclear medicine	260	109.1	13.8
	Pathology	963	2.8	50.6

OB/GYN = obstetrics and gynecology; M KRW, million Korean won. Reimbursement threshold (vertical dashed line in Figure 1) = median NHI-covered reimbursement across 26 specialties. Application-rate threshold (horizontal dashed line) = 100% application rate (slots exactly filled). Q1 = above-median reimbursement, ≥100% fill; Q2 = above-median reimbursement, <100% fill; Q3 = below-median reimbursement, ≥100% fill; Q4 = below-median reimbursement, <100% fill. Two specialties without residency programs (tuberculosis, preventive medicine) excluded.

**Table 5:** Spearman rank correlation between NHI-covered reimbursement per specialist and residency application rate.

Analysis	Year	*n*	*ρ*	95% CI	*p* value
All 24 specialties	2023	24	0.180	−0.24 to 0.54	0.400
All 24 specialties	2024	24	0.101	−0.32 to 0.48	0.639
Excluding DPS*	2023	21	0.281	−0.17 to 0.64	0.218

CI, confidence interval; DPS, dermatology, plastic surgery, ophthalmology. *DPS = dermatology, plastic surgery, and ophthalmology (high non-NHI revenue specialties). Two-tailed *p* < 0.05 defined significance. No coefficient reached this threshold. At *n* = 24, post-hoc power for detecting *ρ* = 0.40 (*α* = 0.05) was about 49%, implying a 51% type II error rate.

### 3.5 Sensitivity analysis

Three specialties with large non-NHI revenue streams were examined separately: dermatology (Q3; application rate 158.6%), plastic surgery (Q3; 161.1%), and ophthalmology (Q1; 175.5%). Dermatology and plastic surgery had below-median NHI reimbursement yet attracted high trainee demand. Ophthalmology had above-median NHI reimbursement (936.7 million KRW) and was classified in Q1, but elective refractive surgery constitutes a substantial share of its practice income. All three derive revenue disproportionately from non-NHI services. After removing these three (*n* = 21), *ρ* increased to 0.281 (95% CI, −0.17 to 0.64; *p* = 0.218; Table 5). The *p* value was still above 0.05. Non-insured income, malpractice risk, and on-call frequency seem to affect residency selection more than NHI reimbursement does.

### 3.6 Counterfactual reallocation simulation

Mean NHI-covered reimbursement of Q3/Q4 (source) specialties: 220.5 million KRW per specialist. Mean of Q2 (destination) specialties: 1,203.6 million KRW. Difference: 983.1 million KRW per head (Table 6). Under 70% efficiency, estimated annual NHI output gains, an upper-bound illustration, were 34.4 billion KRW (50 specialists relocated), 68.8 billion (100), 137.6 billion (200), and 344.1 billion (500). Under 50% efficiency, gains ranged from 24.6 billion to 245.8 billion KRW across the same scenarios.

**Table 6:** Upper-bound illustrative calculation of the NHI output gap between over-supply and shortage quadrants.

Specialists reallocated (n)	100% efficiency (billion KRW)	70% efficiency (billion KRW)	50% efficiency (billion KRW)
50	49.2	34.4	24.6
100	98.3	68.8	49.2
200	196.6	137.6	98.3
500	491.6	344.1	245.8

Source (Q3/Q4) mean reimbursement: 220.5 million KRW. Destination (Q2) mean reimbursement: 1,203.6 million KRW. Marginal NHI gain per specialist: 983.1 million KRW.

NHI, National Health Insurance; KRW, Korean won. Source: mean reimbursement of Q3/Q4 specialties (*n* = 11). Destination: mean of Q2 specialties (*n* = 5). Dermatology, plastic surgery, and ophthalmology have substantial non-NHI revenue; dermatology and plastic surgery are in Q3, whereas ophthalmology is in Q1 and was not included in the Q3/Q4 source mean. Efficiency discounts are arbitrary. These values illustrate the gap, not policy targets.

## 4 Discussion

The inter-specialty gap is large and is not shrinking. Radiation oncology vs. radiology: 50.8-fold. Neurosurgery vs. anesthesiology: 5.3-fold. CV exceeded 90% in both years. A prior national-level analysis published a ratio of medical expenditures per active physician, a single number that, by pooling 26 disciplines into one denominator, concealed the dispersion now documented here (12). Prior to the present analysis, no Korean study had reported NHI reimbursement per specialist at the individual-specialty level. One caveat: NHI reimbursement bundles physician labor with capital-intensive inputs (e.g., linear accelerators in radiation oncology, spinal implants in neurosurgery and orthopedic surgery), and applies facility-tier multipliers that inflate tertiary-hospital claims relative to clinic claims for identical procedures (19, 20). The 50.8-fold figure therefore overstates pure physician labor differences and should be read as reflecting total NHI-financed resource consumption per specialist, not physician effort alone.

The quadrant classification did not change between 2023 and 2024. Q2 included cardiothoracic surgery (51.4% fill rate), general surgery (65.1%), emergency medicine (85.2%), OB/GYN (71.9%), and radiation oncology (73.9%). The average fill rate of Q2 was 69.5%. All five had above-median NHI workloads. When the mean (654.6 million KRW) was substituted for the median as the reimbursement threshold, four of the five remained in Q2; the exception was OB/GYN, whose reimbursement (637.1 million KRW) fell just below the mean. The seven Q3 specialties had a mean application rate of 137.7% on below-median reimbursement; the five core Q3 specialties (excluding occupational-environmental medicine and laboratory medicine) averaged 151.7%. In 2024, pediatrics moved from Q4 to Q2 and emergency medicine moved from Q2 to Q4; the remaining quadrant assignments were unchanged.

Spearman *ρ* was 0.180 (95% CI, −0.24 to 0.54; *p* = 0.400). With only 24 specialties, the statistical power to detect ρ = 0.40 at *α* = 0.05 was approximately 49% (21); the confidence interval spanned both moderate positive and moderate negative values. This null result is uninformative about the direction of any underlying association and is not interpreted as evidence that reimbursement and competition are unrelated. Surveys of medical graduates have reported that non-NHI income, malpractice risk (high in obstetrics and neurosurgery), on-call duty, and work-life balance are the main factors in specialty selection (7, 9, 10, 22). The counterfactual calculation is presented as an upper-bound illustration and not as a policy projection. It assumes that a relocated specialist would generate the mean reimbursement of the destination quadrant, which is internally optimistic because reimbursement embeds capital and practice-expense inputs that a transferring physician would not carry. The efficiency brackets (100, 70, 50%) are arbitrary, as no empirical estimate of retraining loss exists for the Korean setting, and specialist retraining requires 4 to 6 years. The exercise therefore quantifies the magnitude of the inter-quadrant reimbursement gap under the current distribution rather than a realizable policy gain.

Dermatology (158.6% application rate), plastic surgery (161.1%), and ophthalmology (175.5%) attracted far more applicants than their NHI-covered reimbursement would predict. All three derive a substantial share of practice revenue from services outside the NHI fee schedule (aesthetic skin treatments, cosmetic surgery, and elective refractive procedures, respectively). An analysis of Korean relative-value-unit data showed that when NHI fee-schedule valuations are compressed, physicians migrate toward non-NHI-oriented fields (23). The term applied to this phenomenon was “balloon effect.” Dermatology and plastic surgery occupy Q3 (below-median NHI reimbursement, above-100% fill); ophthalmology sits in Q1 (above-median NHI reimbursement) yet shares the same non-NHI income dynamic. In all three cases, trainee demand exceeds what NHI-covered reimbursement would predict, and the difference is attributable to non-NHI income that the claims dataset does not capture.

The 2024 departure of about 12,000 trainees did not change the ranking of specialties in any meaningful way (4, 5). What governs the ordering appears to be fee-schedule weights and case-mix, not the workforce shock. Two specialties did not follow this pattern. Pediatrics shifted from Q4 to Q2 as its per-specialist reimbursement rose 7.9% year-on-year, from 572.4 to 617.5 million KRW (specialists: 6,355 to 6,466), crossing above the 2024 median, even though its 2024 first-round application rate, 25.9%, was the lowest of all specialties. The rise in per-specialist reimbursement alongside a near-static headcount and persistently low trainee demand is consistent with a higher workload borne by the existing workforce rather than with increased trainee interest (24). Claims data cannot distinguish real efficiency gain from a simple extension of working hours per pediatrician (25). Per-specialist reimbursement of emergency medicine fell 14.1%, matching the reduced case volume during the trainee-absence period (5, 26).

The present analysis summed NHI reimbursement across all five facility tiers without stratification. Each tier was affected differently by the trainee departure. Residents formed about 38% of physician staff at tertiary and general hospitals but far fewer at clinics, producing tier-specific case volume shifts in 2024 (27). Had the dataset been stratified by tier, reimbursement at large hospitals would fall below the aggregate values and clinic-level reimbursement would rise above them. Tier-stratified analysis is a necessary next step. The 2024 addition of 2,000 medical school seats was introduced without any specialty-specific allocation mechanism for graduates. Q2 shortages are expected to continue in the absence of such steering (11). In several OECD countries, demand-based physician supply projections have been shown to widen distributional imbalance when service utilization is treated as a proxy for need (28).

In the Korean relative-value-unit system, three elements (physician work, practice expense, and malpractice insurance) are packaged into a single per-procedure value with no component-level breakdown (29). Unbundling the three components is one possible policy direction. The per-specialist figure in radiation oncology, neurosurgery, and orthopedic surgery (above-median-reimbursement fields) comes largely from practice expense, not from physician work. Under US Medicare, 195 procedure codes fell below direct cost after equipment spending inflated the practice-expense component. Physician-work values did not change (30). A selective increase in the physician-work component for essential specialties would be more useful for channeling trainees than any uniform fee revision. A uniform increase would mainly enlarge Q1 revenue without filling Q2 vacancies. Because the physician-work component is the element most directly tied to clinical labor, isolating and raising it for shortage specialties, rather than raising the bundled fee, would target the incentive to the labor input that trainees supply while leaving capital and practice-expense components unchanged. Implementing such a change would require the relative-value-unit schedule to report the three components separately, which the current Korean system does not do.

### 4.1 Limitations

Several limitations should be noted. Because the unit of analysis is the specialty (*n* = 26), no inference at the physician level is possible, and subspecialty case-mix differences remain unobserved. The numerator covers NHI revenue only; excluding non-NHI income means that dermatology, plastic surgery, and ophthalmology are understated. The denominator counts all registered specialists, including those in non-clinical roles (administrators, researchers, educators) who file no NHI claims. This analysis used only the first-round application rate. Cardiothoracic surgery and general surgery often fill the remaining positions in later recruitment rounds (15). Two years of data restrict trend assessment. Five-to-ten-year panels would be preferable, yet HIRA’s portal had not posted specialty-level files before 2023 when this analysis was conducted. HIRA data do not currently allow separation of physician-work from practice-expense (29). The metric therefore represents NHI-financed resource consumption per specialist rather than physician effort alone. Finally, the quadrant labels carry normative connotations that the two-year cross-section cannot fully support. The “structural decline” label applied to Q4, and the “critical shortage” and “potential oversupply” labels applied to Q2 and Q3, describe the position of a specialty on the two measured axes in a given year and are not claims about long-term viability. The Q4 label in particular rests on two cross-sections only; a longer panel would be needed to confirm that a low-reimbursement, low-fill position represents a secular trend rather than a transient dip.

## 5 Conclusion

Per-specialist NHI-covered reimbursement differed by 50.8-fold between radiation oncology and radiology; across all 26 specialties the span was wider still, its lower end shaped by the departmental attribution of support-service claims. CV was above 90% in both years. Together these figures suggest a large allocative gap. No statistically detectable rank-order association was observed between trainee competition and NHI workload (*ρ* = 0.180, *p* = 0.400) (21), and this secondary test was underpowered. Even with strong applicant demand, dermatology and plastic surgery were classified in Q3. Ophthalmology received more applicants than its NHI reimbursement would predict. Non-NHI income likely explains the gap, matching the balloon-effect model (23). This study produced three outputs: (1) a specialty-level NHI reimbursement baseline for Korea, (2) a two-axis quadrant framework, and (3) a counterfactual cost estimate under the current specialty distribution. To move beyond these cross-sectional findings, specialty-level data on non-NHI physician income are needed, but HIRA does not publish such data at present.

## Data Availability

Publicly available datasets were analyzed in this study. These data can be found at: https://data.go.kr, https://opendata.hira.or.kr, https://www.mohw.go.kr, and https://kosis.kr.
